# Production of Naturally γ-Aminobutyric Acid-Enriched Cheese Using the Dairy Strains *Streptococcus thermophilus* 84C and *Lactobacillus brevis* DSM 32386

**DOI:** 10.3389/fmicb.2019.00093

**Published:** 2019-02-13

**Authors:** Ilaria Carafa, Giorgia Stocco, Tiziana Nardin, Roberto Larcher, Giovanni Bittante, Kieran Tuohy, Elena Franciosi

**Affiliations:** ^1^Research and Innovation Centre, Food Quality and Nutrition Department, Fondazione Edmund Mach (FEM), San Michele all’Adige, Italy; ^2^Department of Agronomy, Food, Natural Resources, Animals and Environment (DAFNAE), University of Padova, Legnaro, Italy; ^3^Technology Transfer Centre, Fondazione Edmund Mach (FEM), San Michele all’Adige, Italy

**Keywords:** lactic acid bacteria, model cheese, GABA-enriched cheese, health-promoting bacteria, MiSeq Illumina, Ultra High Performance Liquid Cromatography - Orbitrap Q-Exactive Mass Spectrometry

## Abstract

The cheese-derived strains *Streptococcus thermophilus* 84C isolated from Nostrano cheese, and *Lactobacillus brevis* DSM 32386 isolated from Traditional Mountain Malga cheese have been previously reported as γ-aminobutyric acid (GABA)-producers *in vitro*. In the present study, the ability of these strains to produce GABA was studied in experimental raw milk cheeses, with the aim to investigate the effect of the culture and the ripening time on the GABA concentration. The cultures used consisted on *S. thermophilus* 84C alone (84C) or in combination with *L. brevis* DSM 32386 (84C-DSM). The control culture was a commercial *S. thermophilus* strain, which was tested alone (CTRL) or in combination with the *L. brevis* DSM 32386 (CTRL-DSM). The pH evolution, microbiological counts, MiSeq Illumina and UHPLC-HQOMS analysis on milk and cheese samples were performed after 2, 9, and 20 days ripening. During the whole ripening, the pH was always under 5.5 in all batches. The concentration of GABA increased during ripening, with the highest content in 84C after 9 days ripening (84 ± 37 mg/kg), in 84C-DSM and CTRL-DSM after 20 days ripening (91 ± 28 and 88 ± 24 mg/kg, respectively). The data obtained support the hypothesis that *S. thermophilus* 84C and *L. brevis* DSM 32386 could be exploited as functional cultures, improving the *in situ* bio-synthesis of GABA during cheese ripening.

## Introduction

Milk and dairy products are a good food source of high-quality proteins, minerals and vitamins. Because of the presence of saturated fatty acids, some people believe that dairy foods may be detrimental to health, and limit or exclude dairy foods from their diet, especially if they are overweight or predisposed to cardiovascular disease (Rozenberg et al., 2016). However, observational evidences do not support the hypothesis that dairy fat contributes to obesity, and its relation with the increase of low-density lipoprotein cholesterol and development of cardio-vascular disease is still unclear (Muehlhoff et al., 2013).

GABA is a non-protein amino acid acting as inhibitory neurotransmitter in the mammalian central nervous system. GABA has proven effects on brain function, preventing or alleviating anxiety, depression, sleeplessness, memory loss; it stimulates the immune system, prevents inflammation processes, hypertension and diabetes, and regulates the energy metabolism (Dhakal et al., 2012). GABA is naturally present in small quantities in many vegetal foods, and at high concentration in fermented products, especially fermented dairy products and soy sauces (Diana et al., 2014). The concentration of GABA detected in 22 different Italian cheese varieties ranged between 0.260 and 391 mg/kg (Siragusa et al., 2007), and between 320 and 6773.5 mg/kg in Cheddar cheese (Wang et al., 2010; Pouliot-Mathieu et al., 2013). Several authors reported the ability of selected lactic acid bacteria (LAB) and bifidobacteria to produce this GABA *in vitro* from the precursor L-glutamic acid (Siragusa et al., 2007; Li and Cao, 2010; Carafa et al., 2015; Franciosi et al., 2015), and investigated the GABA-producing ability of LAB belonging to *S. thermophilus*, *L. plantarum*, *L. paracasei*, *L. delbrueckii subsp. bulgaricus*, and *Lactococcus lactis* in fermented cows’ milk and yogurt. The GABA concentration detected in the latter studies ranged between 15 and 5000 mg/kg, even though L-glutamate was added to milk before starting the fermentation process (Siragusa et al., 2007; Lacroix et al., 2013; Nejati et al., 2013; Linares et al., 2016). i.e., we reported the ability of *Lactobacillus brevis* BT66 (hereafter DSM 32386) and *Streptococcus thermophilus* 84C isolated from traditional alpine cheeses, to produce high concentration of GABA (Carafa et al., 2015; Franciosi et al., 2015). In the present study, the hypothesis that both strains (*S. thermophilus* 84C as starter and *L. brevis* DSM 32386 as non-starter) are able to produce GABA in cheese and to increase the concentration of GABA over ripening (2, 9, and 20 days) was tested. The use of raw milk was chosen for enhancing the natural production of free amino acids (including L-glutamate) by the proteolytic activity of milk resident bacteria on the peptides released by the hydrolytic action of the calf rennet on caseins (McSweeney, 2004).

To our knowledge, this is the first research addressed to the production of naturally GABA-enriched raw milk cheese, where the effect of two GABA-producing strains and the ripening time are considered as GABA producing factors.

## Materials and Methods

### Microorganisms and Inoculum Preparation

The strains *S. thermophilus* 84C and *L. brevis* DSM 32386 belong to the culture collection of the Department of Food Quality and Nutrition - Fondazione Edmund Mach (FEM, San Michele all’Adige, TN, Italy). Both strains were isolated from traditional alpine cheeses (Nostrano and Traditional Mountain Malga cheese, respectively) and were phenotypically, genotypically, and technologically characterized in previous studies (Carafa et al., 2015; Franciosi et al., 2015). Both strains showed the ability to produce GABA *in vitro*, which was the reason for interest in the present study.

The commercial *S. thermophilus* was isolated and purified from the commercial lyophilized starter mix culture LYOFAST MOT 086 EE (Sacco, Cadorago, CO, Italy), which did not show any GABA-producing activity *in vitro*, and was selected as control strain. The *S. thermophilus* 84C and the commercial *S. thermophilus* were grown in M17 broth at 45°C, and *L. brevis* DSM 32386 was grown at 30°C in de Man, Rogosa and Sharpe (MRS) broth, acidified to pH 5.5 with lactic acid 5 mol/L. After 48 h incubation, the strains were individually sub-cultured in M17 or MRS (1%, v/v) and grown for 24 h at their optimal temperature. Cultures were harvested by centrifugation at 3,220 g for 10 min at 4°C, the pellets were suspended in Skim Milk (SM, Oxoid; 10:1, v/v; e.g., 400 mL pure culture in 40 mL SM), divided in 1 mL aliquots and frozen in liquid nitrogen. The cellular concentration of each culture was calculated in triplicate by plate counting onto M17 agar for *S. thermophilus* strains and MRS for *L. brevis*. All media were purchased from Oxoid (Milan, Italy).

**FIGURE 1:**
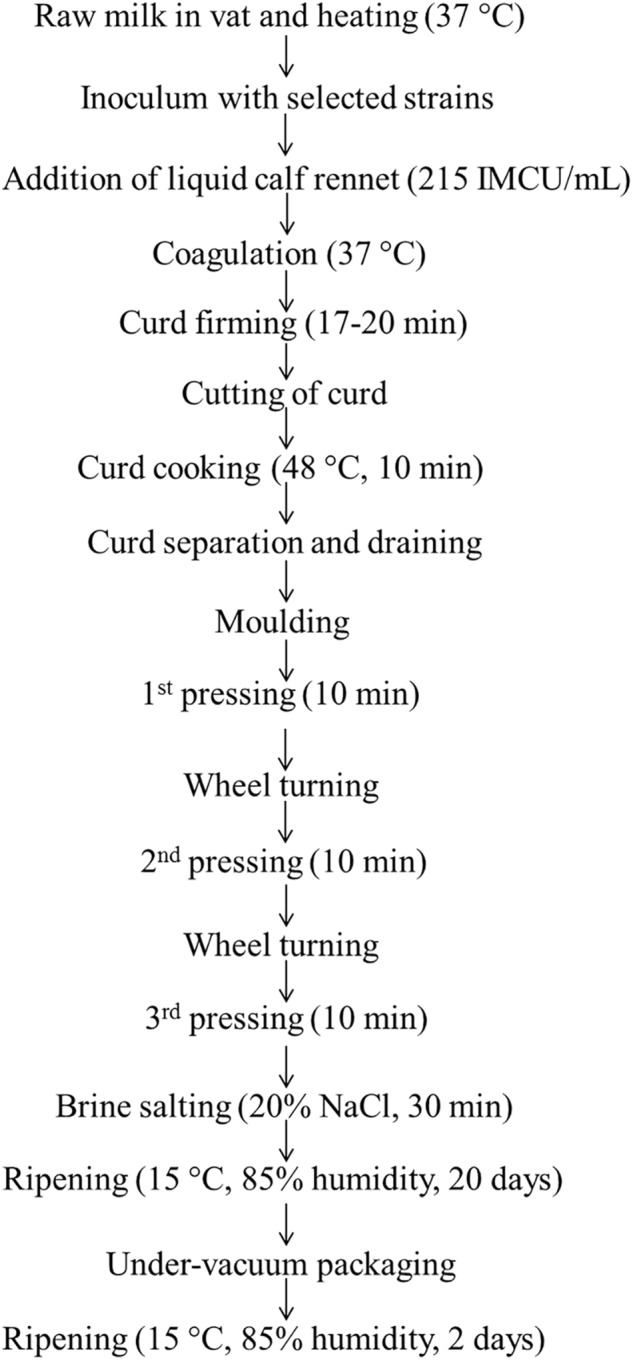
Description of the model cheese-making process.

### Experimental Cheese Manufacture

We produced four mini-cheese batches, as follows: the control (CTRL) mini-cheese was manufactured inoculating raw cow’s milk with the commercial *S. thermophilus* starter strain; the 84C mini-cheese (84C) was produced inoculating *S. thermophilus* 84C as starter strain; the CTRL-DSM mini-cheese was produced adding the commercial *S. thermophilus* as starter strain and *L. brevis* DSM 32386 as adjunct culture, and the 84C-DSM mini-cheese was produced inoculating *S. thermophilus* 84C as starter and *L. brevis* DSM 32386 as adjunct culture. Both commercial and 84C *S. thermophilus* strains were inoculated into bulk milk at concentration 10^6^ CFU/mL, and *L. brevis* DSM 32386 at concentration 10^1^ CFU/mL. Experimental cheeses were produced according to the method described by Cipolat-Gotet et al. (2013), with some modifications (Figure 1). Raw cow’s milk (1.5 L) was heated at 37°C, inoculated with the bacterial mixture and coagulated with the addition of calf rennet (Naturen Plus 215 Hansen, Pacovis Amrein AG, Bern, Switzerland, 215 IMCU/mL). After coagulation, curd was cut into nut-size grains, cooked at 48°C for 10 min, and finally rested at this temperature for 20 more min. After separation, draining and molding, curd was pressed for 30 min at room temperature and salted for 30 min in a saturated brine solution (17°Be, 9°SH/50, 28°C). After two days ripening at 15°C and 85% relative humidity, all batches were stored under-vacuum and ripened for 18 more days. For each batch were produced three wheels to be opened and analysed after 2, 9 and 20 days ripening, and three replicates for each time point were produced in three consecutive weeks for a total of 36 mini-cheese batches. The chemical composition analysis of milk was performed by MilkoScan^TM^ FT6000 (Foss Electric A/S, Hillerød, Danimarca), while the chemical composition of whey and cheese was determined by the FoodScan^TM^ Lab (Foss Electric A/S).

### Microbiological Analysis

Milk, curd, and cheese samples were submitted to microbiological analysis. Four grams of cheese were homogenized with 36 g of sterile Na-citrate 2% (w/w) solution by ULTRA-TURRAX^®^ (IKA^®^ Werke GmbH & Co., KG, Staufen, Germany) for 5 min at 15,000 rpm, inside the microbiological cabinet. Then, milk and cheese samples were decimally diluted and plated onto selective agar media and incubated as follows: MRS agar acidified to pH 5.5 with 5 mol/L lactic acid, anaerobiosis, 48 h at 30°C and 45°C for mesophilic and thermophilic LAB rod-shaped, respectively; M17 agar for 48 h, aerobiosis at 30°C and anaerobiosis at 45°C for mesophilic and thermophilic LAB cocci-shaped, respectively; violet red bile agar (VRBA) for 24 h, aerobiosis at 37°C for coliforms; plate count agar (PCA) with SM (10 g/L, w/v) for 24 h, aerobiosis at 30°C for total bacterial count (TBC). All culture media were purchased from Oxoid.

### DNA Extraction and MiSeq Library Preparation

Illumina analysis was performed on all milk and cheese samples. Ten mL of milk and homogenized cheese samples were centrifuged at 3,220 g for 15 min at +4°C. The genomic DNA was extracted from the pellet using the Power Food^TM^ Microbial DNA Isolation Kit (Mo Bio Laboratories Inc., Carlsbad, CA, United States) according to the manufacturer’s instructions. All DNA samples were purified by PowerClean DNA Clean-up Kit (Mo Bio Laboratories Inc.) and quantified by Nanodrop8800 Fluorospectrometer (Thermo Scientific, United States).

A 464-nucleotide sequence of the bacterial V3-V4 region (Baker et al., 2003; Claesson et al., 2010) of the 16S rRNA gene (*Escherichia coli* positions 341 to 805) was amplified. Unique barcodes were attached before the forward primers to facilitate the pooling and subsequent differentiation of samples. To prevent preferential sequencing of smallest amplicons, the amplicons were cleaned using the Agencourt AMPure kit (Beckman coulter) according to manufacturer’s instructions. The DNA concentration of amplicons was determined using the Quant-iT PicoGreen dsDNA kit (Invitrogen) following the manufacturer’s instructions. In order to ensure the absence of primer dimers and to assay the purity, the generated amplicon libraries quality was evaluated by a Bioanalyzer 2100 (Agilent, Palo Alto, CA, United States) using the High Sensitivity DNA Kit (Agilent). Following quantitation, the cleaned amplicons were mixed and combined in equimolar ratios. Pair-end sequencing was carried out at CIBIO (Center of Integrative Biology) – University of Trento (Trento, Italy) using the Illumina MiSeq system (Illumina, United States).

### Illumina Data Analysis and Sequences Identification by QIIME2

Raw paired-end FASTQ files were demultiplexed using idemp^2^ and imported into Quantitative Insights Into Microbial Ecology (Qiime2, version 2018.2). Sequences were quality filtered, trimmed, de-noised, and merged using DADA2 (Callahan et al., 2016). Chimeric sequences were identified and removed via the consensus method in DADA2. Representative sequences were aligned with MAFFT and used for phylogenetic reconstruction in FastTree using plugins alignment and phylogeny (Price et al., 2009; Katoh and Standley, 2013). Alpha and beta diversity metrics were calculated using the core-diversity plugin within QIIME2 and emperor (Vazquez-Baeza et al., 2013). Taxonomic and compositional analyses were conducted by using plugins feature-classifier^3^. A pre-trained Naive Bayes classifier based on the Greengenes 13_8 99% Operational Taxonomic Units (OTUs) database^4^, which had been previously trimmed to the V4 region of 16S rDNA, bound by the 341F/805R primer pair, was applied to paired-end sequence reads to generate taxonomy tables.

The data generated by Illumina sequencing were deposited in the NCBI Sequence Read Archive (SRA) and are available under Ac. PRJNA497423.

### Quantification of GABA and Glutamate

The amino acid composition of milk and all cheese samples was quantified by Ultra High Performance Liquid Cromatography - Orbitrap Q-Exactive Mass Spectrometry (UHPLC-HQOMS; UHPLC Ultimate 3000RS, ThermoScientific, Rodano, Italy), at the Technology Transfer Centre, Fondazione Edmund Mach (FEM, San Michele all’Adige, Italy).

Two grams of cheese were mixed to 0.4 g of sulfosalicylic acid, suspended in 29.7 mL of perchloric acid (0.01 M) and 0.3 mL of β-glutamic acid (500 mg/L) and homogenized with a ULTRA-TURRAX^®^ for 10 min at 15,000 rpm. The suspensions were submitted to sonication for 30 min and centrifuged at 3,220 g for 20 min. The supernatant was filtered through a 0.22 μm pore size filter (Minisart, Sartorius Stedim Biotech, Goettingen, Germany) and diluted 1:50 with a water/methanol solution (50:50, *v/v*). For milk, 20 grams of sample were mixed to 0.4 g of sulfosalicylic acid, cooled in ice for 10 min and centrifuged at 3,220 g for 10 min. Two grams of supernatant were suspended in 29.7 mL of perchloric acid (0.01 M) and 0.3 mL of β-glutamic acid (500 mg/L) and homogenized. Samples were filtered through a 0.22 μm filter (Minisart, Goettingen) and diluted 1:50 with a water/methanol solution (50:50, *v/v*).

The separation was carried out with formic acid 0.1% (v/v; eluent A; Sigma) and methanol with formic acid 0.1% (v/v; eluent B; Sigma), injecting 5 μL of sample through an Acclaim Trinity P1 column (3 μm particle size, 100 mm × 2.1 mm I.D.; Merk, Germany) at 35°C. The flow rate was set at 0.4 mL/min. The analytical gradient for eluent B was: 1 min at 2%, 4 min at 30%, up to 50% in 0.5 min, to 100% in 0.5 min, held at 100% for 3 min for cleaning, and to 2% for reconditioning in 0.5 min.

Mass spectra were acquired in positive mode through a full MS analysis at mass resolving power 70,000. For ionization, HESI II parameters were set as follow: heated capillary temperature to 330°C; sheath gas flow rate at 40 arbitrary units; auxiliary gas flow rate at 20 arbitrary units; spray voltage at 3.0 kV; auxiliary gas heater temperature at 300°C.

GABA, glutamic acid and the internal standard β-glutamic acid were detected in the extracted ion chromatograms (EICs) corresponding to the protonated molecules [M+H]^+^ (mass tolerance < 5 ppm) used for quantification, whereas dd-MS/MS spectra compared with those collected from available standards were used for confirmation.

Calibration curves were obtained by plotting the peak area ratio of the quantifier ions (A_standard_/A_internalstandard_), multiplied by the internal standard concentration, versus the corresponding concentration level. Precision, expressed as Relative Standard Deviation (RSD%) of repeatability was tested injecting the same sample four times within the sequence and must be ≤20% (SANTE guidelines 11945, 2015).

### Statistical Analysis

One-way analysis of variance (one-way ANOVA) was performed on all data using STATISTICA data analysis software system, version 13 (TIBCO Software Inc., 2017). Multiple comparison of means was performed using Tukey’s test at a *p* value of <0.05.

**FIGURE 2:**
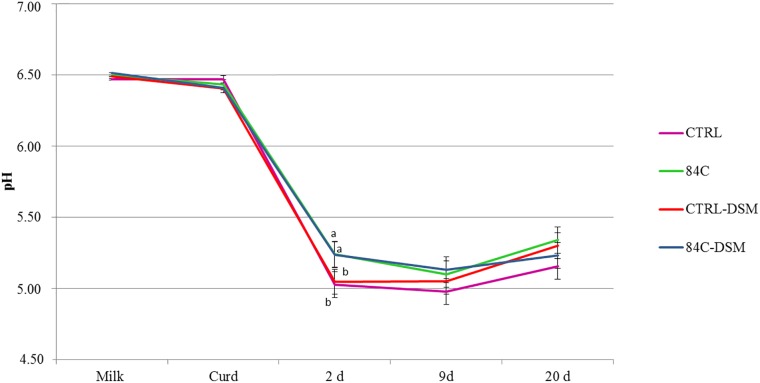
pH dynamic of the experimental cheeses during ripening. Values are expressed as mean value ± standard deviation. Different letters (a–c) indicate a significant difference (*p* < 0.05) among the batches at the same sampling point (Tuckey’s test) indicate significant differences.

## Results

### Physico-Chemical Characteristics and Microbial Counts of the Experimental Cheeses During the Ripening Time

The physico-chemical characteristics of milk, whey and cheese after 20 days of ripening are shown in Table 1. The different batches did not show any significant difference in terms of chemical composition (*p* > 0.05). The pH evolution was recorded in milk and during the cheese making process, as shown in Figure 2. The pH value of milk ranged between 6.47 and 6.51 and slightly decreased in curd after extraction with no significant differences among the four batches (6.41–6.47, *p* > 0.05). After 2 days ripening a further reduction of pH was observed in all batches, and cheese samples manufactured with the commercial *S. thermophilus* culture showed a significantly lower pH (5.05) than the cheeses produced with the cultures including *S. thermophilus* 84C (5.24). The lowest pH value was reached after 9 days ripening, with no significant differences (*p* > 0.05) among the four batches (the pH ranged between 4.98 and 5.13). At the end of the ripening period, the pH slightly increased to 5.16–5.34 with no significant differences (*p* > 0.05) among the cheese batches studied.

**Table 1:** Chemical composition of milk, whey and cheese used in the experimental cheese production.

	MV	*SD*
**Milk**		
Fat, %	3.58	0.08
Protein, %	3.24	0.02
Lactose, %	5.10	0.01
Caseins, %	2.64	0.02
Total solids, %	12.49	0.09
pH	6.50	0.02
SCS^1^, units	3.33	0.31
**Whey**		
Fat, %	0.66	0.07
Protein, %	0.87	0.02
Lactose, %	5.08	0.02
pH	6.46	0.03
**Cheese**		
Weight, g	119.18	16.29
Fat, %	32.70	4.49
Protein, %	25.71	2.58
Salt, %	2.04	0.07
Total solids, %	69.73	9.94
pH	5.30	0.32
Moisture, %	29.92	5.95
FDM^2^, %	47.21	2.30

*^1^SCS: somatic cell count = 3+log_2_(SCC/100). ^2^FDM: fat dry matter.*

The total bacterial counts in milk, curd, and experimental cheeses after 2, 9, and 20 days ripening are shown in Table 2.

**Table 2:** Changes in microbial counts of milk and cheese batches throughout ripening (curd, 2, 9, and 20 days).

	TBC		M17 30°C		M17 45°C		MRS 30°C		VRBA	
	MV	*SD*	MV	*SD*	MV	*SD*	MV	*SD*	MV	*SD*
**Milk**	5.4^a^	0.8	5.0^a^	0.5	0.4^a^	1.0	4.1^a^	0.3	1.5^a^	0.6
**CTRL**										
curd	7.6^b^	0.9	7.1^b^	0.3	7.2^b^	0.2	7.2^b^	0.3	1.6^a^	0.9
2 days	8.9^c^	0.1	8.7^c^	0.2	8.3^b,c^	0.2	9.0^c^	0.2	4.9^b,c^	1.5
9 days	8.1^b,c^	0.3	9.0^c^	1.1	8.0^b,c^	0.1	8.2^b,c^	0.4	4.7^b^	0.6
20 days	8.9^c^	0.8	7.7^b,c^	1.2	7.1^b^	0.7	8.3^b,c^	1.6	4.4^b^	0.9
**84C**										
curd	7.6^b^	0.6	7.2^b^	0.3	7.4^b^	0.2	7.3^b^	0.3	2.0^a^	1.1
2 days	8.6^c^	0.4	8.6^c^	0.4	7.9^b,c^	0.1	8.9^c^	0.3	4.4^b^	0.4
9 days	7.9^b^	0.3	7.7^b,c^	0.1	7.1^b^	0.4	8.0^b,c^	0.2	5.3^c^	1.1
20 days	8.3^b,c^	0.9	7.8^b,c^	0.3	7.4^b^	0.3	8.5^c^	0.9	5.1^b,c^	0.4
**CTRL-DSM**										
curd	7.3^b^	0.4	7.3^b^	0.5	7.2^b^	0.1	7.1^b^	0.3	1.9^a^	1.0
2 days	9.0^c^	0.4	8.8^c^	0.4	8.7^c^	0.3	9.1^c^	0.4	4.5^b^	1.6
9 days	8.4^b,c^	0.2	8.4^b,c^	0.3	7.7^b,c^	0.4	8.2^b,c^	0.4	5.0^b,c^	0.6
20 days	8.0^b,c^	0.7	7.8^b,c^	0.7	7.6^b,c^	0.2	8.3^b,c^	1.6	4.9^b,c^	0.5
**84C-DSM**										
curd	7.8^b,c^	1.1	7.4^b^	0.9	7.4^b^	0.3	7.2^b^	0.3	2.2^a^	0.8
2 days	8.7^c^	0.9	8.6^c^	0.8	7.6^b,c^	0.4	8.4^b,c^	0.8	4.9^b,c^	1.1
9 days	8.5^b,c^	0.2	8.5^c^	0.2	7.9^b,c^	0.4	8.4^b,c^	0.5	5.1^c^	0.5
20 days	8.6^c^	0.5	7.6^b,c^	0.4	7.3^b^	0.3	8.5^c^	1.4	4.9^b,c^	0.7

*The microbial counts in milk were performed before Streptococcus thermophilus (both commercial and 84C) and Lactobacillus brevis DSM 32386. Control (CTRL) was produced adding the commercial S. thermophilus, 84C cheese (84C) was produced inoculating S. thermophilus 84C; DSM cheese (CTRL-DSM) was produced adding the commercial S. thermophilus and L. brevis DSM 32386, and 84C-DSM cheese (84C-DSM) was produced inoculating S. thermophilus 84C and L. brevis DSM 32386. a, b, c, and d: Different letters in the same column indicate significant statistical differences (Tukey’s Test p < 0.05).*

The count of all microbial groups detected in milk increased significantly (*p* ≤ 0.001) in the curd with the exception of coliforms. The highest total bacterial counts were observed after 2 days ripening (8.6–9.0 log CFU/g), with no significant differences among the different batches. After 9 and 20 days ripening, total bacterial counts showed a little decreasing trend with no significant differences (*p* < 0.05) among batches.

LAB (M17 and MRS counts) showed a similar trend of total aerobic bacteria throughout the ripening. LAB counts increased for 2 days, and then decreased approximately one logarithmic cycle (*p* > 0.05) until the end of ripening.

The load on M17 30°C showed a trend similar to TBC, without any significant difference among batches. The bacterial concentration on M17 45°C was very low in bulk milk (<1 log CFU/mL) and increased in curd after inoculating the tested strains, ranging between 7.2 ± 0.2 and 7.4 ± 0.3 log CFU/g. After 2 days ripening, the count on M17 45°C increased by about one logarithmic unit (*p* > 0.05) in cheese samples inoculated with the commercial *S. thermophilus*, and was stable in samples containing the autochthonous *S. thermophilus* 84C. Milk contained 4.1 ± 0.27 log CFU/mL bacteria on MRS, which increased by about 3 and 4–5 logarithmic units in curd and cheese, respectively.

Very low counts on VRBA were detected in milk (1.5 ± 0.6 log CFU/mL), which reached the maximum count in cheese after 9 days ripening (range between 4.7 ± 0.3 and 5.3 ± 1.1 log CFU/g).

### MiSeq Sequencing Data

The sequences obtained by MiSeq Illumina analysis were first submitted to merging and quality trimming, and 1,741,006 reads were subsequently analyzed. After alignment, the remaining Operational Taxonomic Units (OTUs) were clustered at 3% distance. In order to analyze the bacterial community richness in milk and cheese, the number of OTUs and the diversity Shannon index were determined using QIIME 2 at 97% similarity levels. Data are shown in Table 3.

**Table 3:** Number of sequences analyzed (N reads), diversity richness (Chao 1), Observed OTUs (OTUs), and diversity index (Shannon) for experimental cheeses and milk samples.

Sample	N reads	Observed OTUs	Chao1	Shannon
**Milk**	17.167	82	84.05	4.76
**CTRL**				
2 days	50.419	235	272.97	6.43
9 days	47.698	193	209.70	5.99
20 days	37.841	155	190.83	5.41
**84C**				
2 days	52.458	288	331.32	6.58
9 days	46.364	311	362.34	6.25
20 days	47.323	261	313.69	6.24
**CTRL-DSM**				
2 days	51.551	261	296.26	6.49
9 days	46.493	229	260.42	6.45
20 days	48.336	239	279.26	6.10
**84C-DSM**				
2 days	47.072	219	248.95	5.75
9 days	44.886	237	275.37	5.94
20 days	42.727	196	226.01	5.47

**FIGURE 3:**
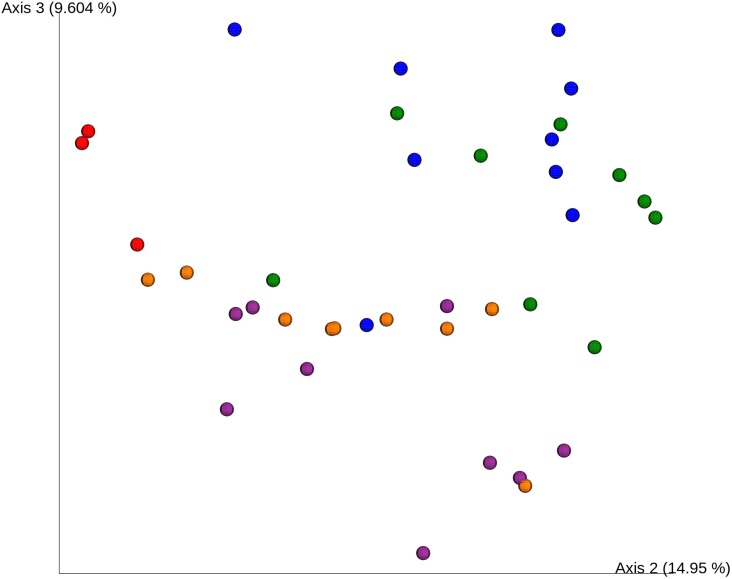
Bray Curtis analysis of milk and cheese samples after 2, 9, and 20 days ripening. Red dots show milk samples (*n* = 3), the blue dots show the CTRL cheese (*n* = 9), green dots show CTRL-DSM cheese (*n* = 9), purple dots show 84C cheese (*n* = 9), and orange dots show 84C-DSM cheese samples (*n* = 9).

The differences between samples were also evaluated by Bray Curtis phylogenetic metric (Figure 3). Cheeses inoculated with the 84C strain (84C and 84C-DSM) clustered together (orange and violet icons) on the third axis and were separated from cheeses inoculated with the commercial *S. thermophilus* starter strain. The PERMANOVA analysis, showed a higher dissimilarity among samples inoculated with different *S. thermophilus* strains (*p* < 0.01) than samples inoculated with or without *L. brevis* DSM 32386.

**FIGURE 4:**
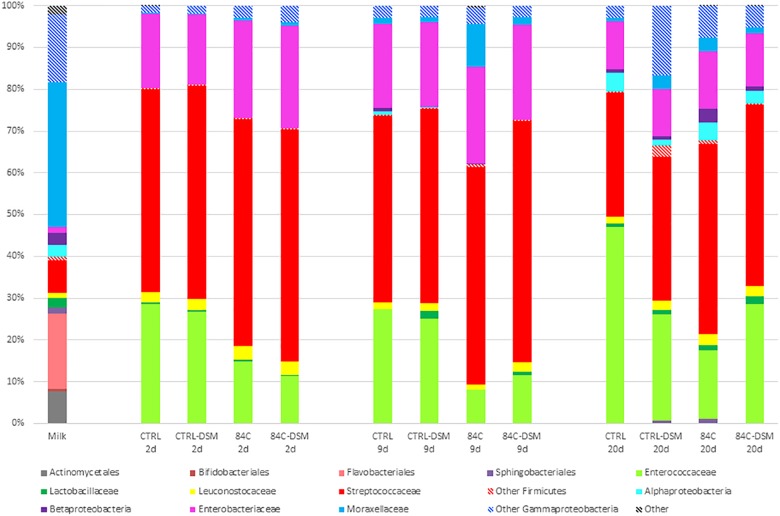
Relative abundance of the microbial taxonomic groups detected in milk and cheese samples after 2, 9, and 20 days ripening. Data are expressed in percentage.

### Microbial Communities Identified in Milk and Cheese Samples

The milk community was mainly constituted by *Moraxellaceae* (35%), *Flavobacteriales* (18%) and other *Gammaproteobacteria* (*Chryseobacterium* and *Pseudomonas*, 16%) (Figure 4). *Streptococcaceae* and *Lactobacillaceae* were detected at 8 and 2% relative abundance, respectively.

The cheese microbiota after 2 days ripening (Figure 4) showed that *Streptococcaceae* was the most abundant family in all batches (49% relative abundance in CTRL, 51% in CTRL-DSM, 54% in 84C and 56% in 84C-DSM). Furthermore, we observed other dominant microbial families as *Enterococcaceae* (29% in CTRL, 27% in CTRL-DSM, 15% in 84C and 11% in 84C-DSM) and *Enterobacteriaceae* (18% in CTRL, 27% in CTRL-DSM, 23% in 84C and 25% in 84C-DSM).

After 9 days ripening the microbial composition did not show significant change, with the exception of cheese samples inoculated with *L. brevis* DSM 32386 strain where *Lactobacillaceae* were detected at 1.9 (CTRL-DSM) and 0.9% (84C-DSM), while in the other cheeses this microbial family was under 0.01% relative abundance.

At the end of ripening there was a change of the microbiota, in particular in cheese samples inoculated with the commercial *S. thermophilus* starter. We observed a significant decrease in *Streptococcaceae* from 45 to 30% and an increase of *Enterococcaceae* from 27 to 47% that became the dominant microbial family in this cheese. *Enterobacteriaceae*, ranging between 11 and 14%, decreased in all batches (Figure 4).

**FIGURE 5:**
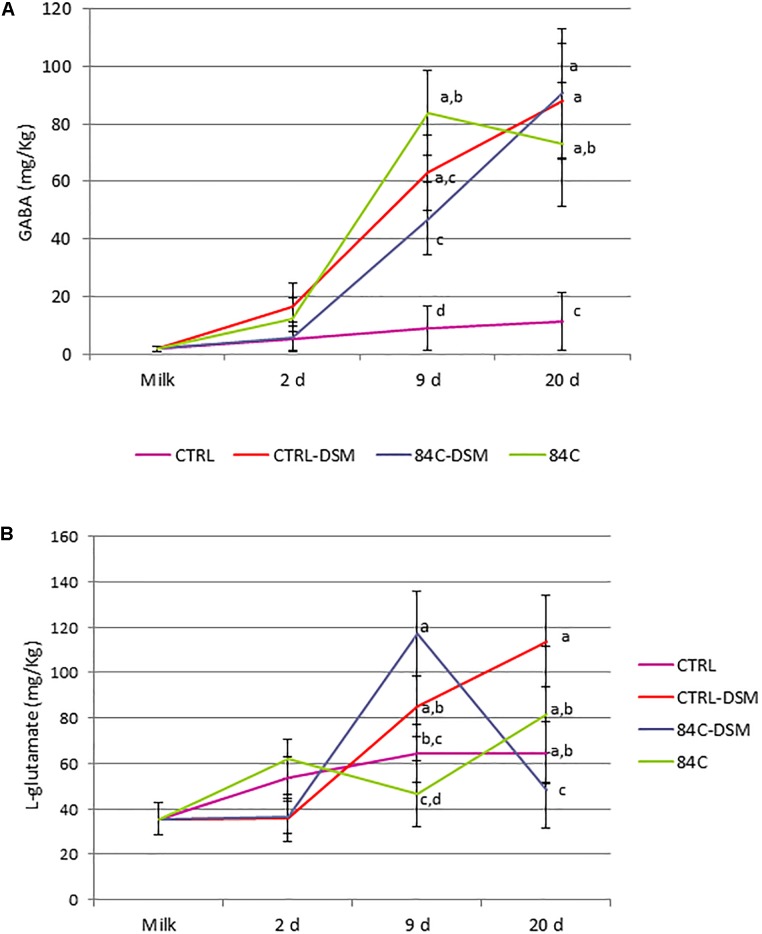
GABA **(A)** and L-glutamic acid **(B)** concentration detected in milk and cheese samples after 2, 9, and 20 days ripening. The mean value ± standard deviation of both amino acids is expressed in mg/kg. Different letters (a, b, c) indicate a significant difference (*p* < 0.05) among the batches at the same sampling point (Tuckey’s test) indicate significant differences.

### GABA Detection in Milk and Cheese Samples

The GABA concentrations are shown in Figure 5A. The UHPLC-HQOMS analysis on milk and cheese samples showed an increase of GABA during ripening, with the highest content in 84C after 9 days ripening (84 ± 37 mg/kg), in 84C-DSM and CTRL-DSM after 20 days ripening (91 ± 28 and 88 ± 24 mg/kg, respectively). Milk contained 1.9 ± 0.9 mg/kg GABA, which slightly increased (*p* > 0.05) in all cheese batches after 2 days ripening. The first significant change was observed in CTRL-DSM, 84C-DSM and 84C samples after 9 days ripening, which contained significantly more GABA (63 ± 19, 47 ± 22, and 84 ± 37 mg/kg, respectively) than CTRL (9.1 ± 7.8 mg/kg, p < 0.05). At the end of ripening (20 days), 84C-DSM cheese had the highest level of GABA (91 ± 22 mg/Kg), which was not significantly different from CTRL-DSM (88 ± 20 mg/Kg) and 84C (73 ± 21 mg/Kg), but significantly higher than CTRL cheese (11 ± 10 mg/Kg).

Glutamic acid was also detected in milk (36 ± 7 mg/kg) and cheese during ripening (Figure 5B). After 2 days ripening the content of glutamate ranged between 36 ± 10 and 62 ± 39 mg/kg, and increased after 9 days ripening between 47 ± 17 and 117 ± 8 mg/kg. We observed a significantly higher content of glutamate in 84C-DSM after 9 days ripening (117 ± 8 mg/kg), compared to CTRL (64 ± 37 mg/kg) and 84C batches (47 ± 17 mg/kg). At the end of ripening, the concentration of glutamate fluctuated between 48 ± 38 and 114 ± 40 mg/kg glutamate, with no significant differences between batches.

## Discussion

In the present study, the use of raw milk was chosen in order to exploit the ability of the resident microbiota to release naturally free amino acids (including L-glutamate) from the peptides originated from the hydrolytic action of the calf rennet on caseins (McSweeney, 2004).

The experimental mini-cheese batches were produced in the laboratory, developing a cheese making protocol which could simulate as better the technology of alpine raw milk cheeses in very small vats. The processing of small volumes of milk (1.5 L) has the advantage that up to 20 cheese batches/day can be produced using the same milk, but the disadvantage that the wheels are very small, and the high surface/volume ratio makes the cheese subjected to a fast drying process. For this reason the ripening process was conducted under-vacuum in order to mimic the anaerobic conditions of the cheese core and avoiding the formation of thick rinds.

All cheese batches had similar chemical composition at the end of ripening, suggesting that the tested strains operated similarly.

The pH evolution was monitored during the cheese making process because the role of pH is not only related to the outcome of the fermentation, but also to the production of GABA. The commercial *S. thermophilus* reduced the pH of cheese in a shorter time than the strain *S. thermophilus* 84C within 2 days ripening. Conversely, any of the tested strain (both alone and in combination with *L. brevis* DSM 32386) had not statistically significant effect (*p* > 0.05) on pH after 9 and 20 days ripening.

With regards to the microbiological concentration, we observed an increase of the plate counts in curd samples for all microbial batches, which is probably due to the physical retention of bacteria in the curd and to the multiplication of the inoculated strains during the coagulation phase. The addition of *L. brevis* DSM 32386 was not detectable on MRS 30°C, and it might be related to the initial concentration of lactobacilli in raw milk (4.1 log CFU/mL), which was similar to the concentration of the inoculated *L. brevis.*

Analyzing the sequences obtained by MiSeq Illumina, we obtained much information looking at the quality of the reads and alpha and beta-diversity. The rarefaction analysis based on OTUs at 97% similarity showed approximation to asymptote, and we concluded that the recovered sequences represented properly the diversity of the bacterial communities in the 39 samples.

Based on the OTUs number and the Shannon and Chao indexes, there was no difference related to the inoculated strain. There was a decreasing richness and diversity of microbial population with fermentation time. In fact, the number of OTUs and Chao and Shannon indexes were higher in samples collected after 2 than after 20 days but these differences were not significant. The presence or absence of *L. brevis* DSM 32386 did not significant influence the Bray Curtis index, as also confirmed by PERMANOVA analysis.

Both alpha- and beta-diversity analysis suggested that *L. brevis* had no effect on the microbiota of experimental cheeses at the concentration used in this work. Furthermore, the differences in cheese microbiota are likely due by the starter used even if belonging to the same species and added at the same concentration.

The presence in milk of *Chryseobacterium* and *Pseudomonas* which are gram-negative spoilage bacteria was not unexpected because they are usually present in raw milk and are able to grow at low temperature during refrigerated storage (Quigley et al., 2013; von Neubeck et al., 2015). Conversely, *Streptococcaceae* and *Lactobacillaceae*, which are involved in dairy fermentation and in the determination of organoleptic, flavor and texture properties of the final product (Quigley et al., 2013) are totally desired. The dominance of *Streptococcaceae* after 2 days ripening suggested that bacteria belonging to this family (including the added *S. thermophilus* strains) started the fermentation process. After 9 days ripening *Lactobacillaceae* were significantly higher in CTRL-DSM and 84C-DSM cheeses, suggesting that the sequences identified as *Lactobacillaceae* corresponded to the inoculated *L. brevis* DSM 32386. Taxonomy data at the end of ripening suggested that only the commercial *S. thermophilus* starter strain influenced significantly the cheese microbiota and that all the tested strains had a decreasing effect on the relative abundance of *Enterobacteriaceae*.

During cheese ripening, L-glutamate which is naturally present in caseins (Zoon and Allersma, 1996) might be released from caseins proteolysis. In cheese, the ripening period can facilitate this process, and L-glutamate can be converted to GABA by GABA-producing bacteria. For this reason we chose cheese as GABA-carrier food, even though many factors play a key role on the final content of GABA, like as the cheese-making process, the presence of starter or adjunct cultures and the ripening conditions (Siragusa et al., 2007). In order to determine when GABA is produced and if it persist during ripening, we decided to monitor the content of GABA at different ripening stages. We detected very interesting variations of GABA and glutamate between the four batches. High production of GABA by fermentation is correlated to the activity of glutamic acid decarboxylase (GAD) but also on the inhibition of GABA-decomposing enzymes. GABA transaminase (GABA-T) promotes the reversible conversion of GABA to succinic semi-aldehyde using either pyruvate-dependent GABA transaminase (GABA-TP) or α-ketoglutarate-dependent GABA transaminase (GABA-TK), and succinic semi-aldehyde dehydrogenase catalyzes the reversible conversion of succinic semi-aldehyde to succinate (Takayama and Ezura, 2015).

Overlapping GABA and glutamate concentration we hypothesized that the presence of *L. brevis* DSM 32386 enhanced the release of glutamate from caseins between day 2 and day 20, and the consequent conversion of L-glutamate to GABA. In fact, the increase of glutamate in CTRL-DSM samples after 9 and 20 days ripening corresponded to the increase of GABA. These results suggested that glutamate was gradually consumed over its release and converted to GABA.

By contrast, *S. thermophilus* 84C showed a different trend; the GABA production significantly (*p* < 0.05) increased in 84C cheese samples between 2 and 9 days ripening, but it was related to a slight reduction of L-glutamate. Afterwards, between day 9 and day 20, we observed a slight decrease of GABA and an increase of L-glutamate 84C samples. These data suggest that the strain *S. thermophilus* 84C is likely not involved in the release of caseins, and that GABA is metabolized to succinate and reconverted to L-glutamate after reentering the Krebs cycle. This hypothesis is supported by 84C-DSM cheese samples, where we observed a constant increase of GABA over all the ripening period and a strong and significant decrease (*p* < 0.05) of L-glutamate between 9 and 20 days ripening. Since we did not perform any metatranscriptome or gene expression analysis, we are not able to confirm this hypothesis, and more investigation needs to be done.

Several studies demonstrated that GABA can reduce high blood pressure in animals and humans, as reviewed by Diana et al. (2014). In human intervention trials, a 12 weeks treatment with 100 ml of fermented milk containing between 10 and 12 mg of GABA, or 50 g of GABA-enriched cheese containing 16 mg of GABA, decreased blood pressure in hypertensive patients (Inoue et al., 2003; Pouliot-Mathieu et al., 2013). The experimental cheese produced in the present study contained about 117 mg/kg, thus a portion of 100 g would provide about 11.7 mg of GABA, covering the total intake needed in order to detect positive effects on human health.

The strains tested in the present study would be useful for the production of bioactive traditional alpine cheeses, which are mostly produced from raw milk. Cheese-makers from Trentino Alps are strongly faithful to tradition, and they are not willing to make any change that could compromise or modify the biodiversity and sensory characteristics of their cheeses. Whereas, they usually agree when they are suggested to use autochthonous strains that are able to improve the product. In this contest, a naturally GABA enriched cheese produced from raw alpine milk would satisfy the needs of both producers and consumers, who increasingly ask for healthy foods, and the marketing of local dairy products would rise. On the other hand, the utilization of raw milk might affect the production of GABA in the experimental cheeses, through a synergistic effect between the indigenous microbiota and the tested GABA-producing strains. From an industrial point of view, the use of pasteurized milk would facilitate the optimization of the GABA production in cheese, the standardization and reproducibility of results. For this reason, a new project aiming to develop a naturally GABA-enriched cheese from pasteurized milk is in progress.

## Conclusion

The aim of this study was to demonstrate that GABA is produced in raw milk cheese and accumulates during ripening. The data obtained showed that after 20 days ripening, 84C-DSM, CTRL-DSM and 84C cheeses had a GABA content significantly higher than CTRL cheese, confirming the hypothesis that *S. thermophilus* 84C and *L. brevis* DSM 32386 could be exploited as bio-functional cultures, facilitating the *in situ* bio-synthesis of GABA during cheese ripening, and providing an option to replace chemical GABA with natural GABA.

We need to take into account that the production of experimental mini-cheeses had some limitations, which are the size of the wheels and the ripening conditions. The ripening took place in aerobic condition only during the first two days ripening and from day 3 to day 20 under-vacuum. The small size allowed producing many cheese batches within the same cheese-making process, while the storage and ripening under-vacuum had the double purpose of miming the anaerobic conditions of the cheese core and avoiding the total drying of cheese. However, this is the first study addressed to the manufacture of GABA-enriched raw milk cheese by LAB and more trials will be carried out in order to optimize the production and the accumulation of GABA in cheese and reduce as much as possible its degradation.

## Data Availability

Most of the relevant data are included within the manuscript. Any additional raw data supporting the conclusions will be made available by the authors on request, without undue reservation, to any qualified researcher.

## Author Contributions

EF and GB devised the study. IC and EF drafted the manuscript. EF, IC, GS, and TN performed the experiments. EF, GB, RL, and KT provided resources and intellectual input that supported the study.

## Conflict of Interest Statement

The authors declare that the research was conducted in the absence of any commercial or financial relationships that could be construed as a potential conflict of interest.

## Footnotes

1 https://docs.tibco.com

2 https://github.com/yhwu/idemp/blob/master/idemp.cpp

3 https://github.com/qiime2/q2-feature-classifier

4 http://greengenes.secondgenome.com/
